# Does early prostacyclin therapy decrease extracorporeal life support use in infants with congenital diaphragmatic hernia?

**DOI:** 10.1038/s41372-024-01920-8

**Published:** 2024-03-05

**Authors:** Jane Huang, Philippe Friedlich, Molly Crimmins Easterlin

**Affiliations:** 1 Division of Neonatology, Department of Pediatrics, Los Angeles General Medical Center, Keck School of Medicine, University of Southern California, Los Angeles, CA USA; 2 Fetal and Neonatal Institute, Division of Neonatology, Children’s Hospital Los Angeles, Department of Pediatrics, Keck School of Medicine, University of Southern California, Los Angeles, CA USA

**Keywords:** Intestinal diseases, Outcomes research

## Manuscript citation

Ramaraj AB, Rice-Townsend SE, Foster CL, Yung D, Jackson EO, Ebanks AH et al; Congenital Diaphragmatic Hernia Study Group. Association Between Early Prostacyclin Therapy and Extracorporeal Life Support Use in Patients With Congenital Diaphragmatic Hernia. JAMA Pediatr. 2023 Jun 1;177(6):582-589 [1].

## Type of investigation

Retrospective analysis of prospectively collected registry data.

## Question

In patients with congenital diaphragmatic hernia (CDH), what is the association between early initiation of prostacyclin (PGI_2_) therapy within the first week of life and the use of extracorporeal life support (ECLS) and postnatal mortality?

## Methods

### Design

Retrospective cohort study.

### Data source

The Congenital Diaphragmatic Hernia Study Group (CDHSG) registry, a multicenter, international consortium including more than 90 centers from 18 countries, in which all data were prospectively entered by participating centers and verified by CDHSG leadership.

### Patients

*Inclusion Criteria* – Infants with CDH born between January 2007 to December 2019 admitted within the first week of life who had the highest likelihood of presenting with CDH-associated cardiopulmonary compromise. *Exclusion Criteria* – Infants with CDH who presented after the first week of life to minimize confounding from differences in severity of CDH-associated cardiopulmonary compromise on survival and outcomes.

### Exposure and control

The exposure was early prostacyclin use, which was defined as intravenous prostacyclin initiation within the first 7 days of life. The exposed or treatment group consisted of infants who received prostacyclin during the first week of life before ECLS cannulation or who never needed ECLS. They were compared to infants who did not receive prostacyclin during the first week of life or who received prostacyclin the same day or after ECLS cannulation (unexposed or control group). Infants who were started on prostacyclin on the same day as ECLS cannulation were not included in the treatment group and were considered unexposed as the exact timing of prostacyclin administration relative to the timing of ECLS cannulation was not ascertainable from the data.

### Outcomes

*Primary outcome* – ECLS use. *Secondary outcomes* – time to ECLS cannulation, duration of ECLS, which was analyzed as both a continuous variable and as a categorical variable with 5 groups (<7 days, 8–14 days, 15–21 days, >21 days, death while receiving ECLS), and in-hospital mortality.

### Analysis

First, all infants in the full cohort were described and analyzed by treatment received in order to describe the association of prostacyclin therapy with several outcomes across the entire study population. Demographic variables evaluated included gestational age at the time of birth, birth weight, CDH defect size and side, repair status, and infant transfer status. Second, to reduce confounding and make the comparison groups more alike (to estimate “the effect of treatment among the treated”), infants who received prostacyclin during the first week of life before ECLS cannulation or who never received ECLS were matched 1:1 to unexposed infants using propensity scores with nearest neighbor matching. Multivariable logistic and linear mixed-effects models were used to evaluate associations between early prostacyclin use during the first week of life with primary and secondary outcomes in both the full and matched cohorts. Covariates were selected a priori based on previously described risk factors associated with ECLS in infants with CDH (APGAR scores at 1 and 5 min, highest and lowest partial pressure of carbon dioxide (PaCO_2_) during first 24 h of life, degree of pulmonary hypertension on first postnatal echocardiogram, defect size, and calendar year of birth for temporal trends). E-values were used to evaluate the potential implications of unmeasured confounding. Center-specific effects were analyzed with intraclass correlation coefficients (ICC).

## Main results

There were 6437 infants with CDH enrolled in the CDHSG registry between January 2007 to December 2019. Of these, 6227 infants from 88 different centers were admitted within the first week of life; 206 infants received early prostacyclin, and 6021 did not receive early prostacyclin. ECLS was used in 22.2% (46/206) infants who received early prostacyclin versus 27.9% (1682/6021) infants who did not receive early prostacyclin. A matched cohort of 294 infants (147 pairs) from 31 different centers was used in the propensity score analysis; ECLS was used in 23.3% (34/147) infants who received early prostacyclin versus 42.9% (63/147) who did not receive early prostacyclin.

In full cohort analyses, descriptive statistics revealed baseline imbalances in study groups, with infants receiving early prostacyclin having more severe disease, as indicated by the greater frequency of transfer for a higher level of care, higher PaCO_2_ in first 24 h of life, larger CDH defects, greater degree of pulmonary hypertension on initial echocardiogram, and more medications for management. Infants who received early prostacyclin and those who did not were similar in terms of estimated gestational age, birth weight, and side of hernia defect.

Results of full cohort analyses showed that early prostacyclin therapy was associated with decreased odds of receiving ECLS (adjusted odds ratio [aOR], 0.60; 95% CI, 0.37–0.96; ICC 0.24) and increased in-hospital mortality (aOR, 1.59; 95% CI, 1.01–2.56; ICC 0.22). There was no significant difference in time to ECLS initiation or mean ECLS duration between the two groups when ECLS duration was analyzed as a continuous variable. However, when ECLS duration was analyzed as a categorical variable, early prostacyclin therapy was associated with decreased odds of longer ECLS course and decreased odds of death on ECLS (aOR, 0.47; 95% CI, 0.25–0.88).

After nearest neighbor propensity matching, descriptive statistics for the matched dataset showed an improved balance of variables among the two groups (more similar frequency of transfer, degree of pulmonary hypertension on first echo, and number of medications for pulmonary hypertension). Among the propensity-matched cohort, early prostacyclin therapy was associated with decreased odds of receiving ECLS (aOR, 0.39; 95% CI, 0.22–0.68) and decreased ECLS duration as both continuous and categorical variables (aOR, 0.43; 95% CI, 0.19–0.96; *P* < 0.001). There was no significant difference in in-hospital mortality between the two groups (aOR, 0.88; 95% CI, 0.52–1.48; ICC 0.09). Sensitivity analyses revealed an E-value of 2.58 for ECLS use and 2.42 for sub-categorized ECLS duration among infants who received early prostacyclin therapy. ICCs of early prostacyclin therapy and ECLS outcomes of full cohort ranged from 0.00 to 0.24 with highest ICC seen with ECLS use (ICC 0.24).

## Study conclusion

Early intravenous prostacyclin therapy within the first week of life was associated with a decrease in ECLS use and duration among patients with CDH in both unmatched full cohort and propensity matched analyses. Early prostacyclin therapy in infants with CDH may be beneficial and decrease the need for resource-intensive, high-morbidity therapy and ECLS.

## Commentary

CDH is a severe congenital anomaly that occurs in 1 in 2000 to 4000 live births [2, 3]. Despite advances in prenatal diagnosis and medical therapies, early mortality remains high at 30–60% of affected infants [3]. Respiratory failure secondary to impaired in-utero lung development resulting in pulmonary hypoplasia, pulmonary hypertension, and cardiac dysfunction is one of the leading causes of mortality [3, 4].

Postnatal medical management of CDH is directed toward providing respiratory and cardiovascular support until definitive surgical repair. Medical therapies include sedation and neuromuscular blockade, prevention of acidosis, use of pulmonary vasodilators, cardiac support with inotropes and vasopressors, and gentle ventilation [5]. About 30% of infants with CDH-associated cardiopulmonary compromise end up receiving ECLS, which is associated with 2.7 to 4 times higher mortality after adjusting for disease severity compared to those who do not receive ECLS [6]. As the use of ECLS is not without complications and is usually a last resort, optimizing other medical interventions to decrease the use of ECLS and mortality becomes crucial.

Multiple pulmonary vasodilators targeting different pathways involved in CDH-associated pulmonary hypertension (CDH-PH), such as inhaled nitric oxide, milrinone, and sildenafil, have previously been studied [7]. Recently, prostacyclin, a potent pulmonary arterial vasodilator, has been increasingly used in the neonatal population [4]. A limited number of small observational studies have evaluated the use of prostacyclin or its synthetic analogs in neonates and reported improvement in pulmonary hypertension [8, 9]. Prostacyclin has been commonly used in the pediatric and adult population to treat pulmonary arterial hypertension for the last 30 years [4]. Prostacyclin decreases pulmonary arterial pressure by acting on prostacyclin receptors of the endothelial and smooth muscle cells, leading to smooth muscle relaxation and vasodilation [4].

Prior to the current study, there were several small studies that focused on the efficacy and safety of prostacyclin in infants with CDH-PH. Carpentier et al. and Lawrence et al. both reported improvement in pulmonary hypertension [10, 11], while Skarda et al. reported no difference in mortality [12]. Currently, there is no consensus or guideline for the use of prostacyclin or its synthetic analogs in infants with CDH-PH, especially regarding timing of initiation and duration prior to ECLS cannulation [4].

This study by Ramaraj et al. is the first large study evaluating early intravenous prostacyclin use during the first week of life in infants with CDH and the outcomes of ECLS and mortality. It demonstrated a potential benefit of decreased ECLS use and duration with early prostacyclin therapy after adjusting for disease severity with propensity score matching. Overall, this is a well-designed retrospective cohort study that had many strengths, including robust statistical techniques that help to answer different questions regarding early prostacyclin use in the CDH population. Full cohort analyses examined the effects of early prostacyclin use and ECLS outcomes across all infants with CDH after adjusting for a priori selected previously described covariates associated with poorer outcomes. Propensity matching helped to adjust for disease severity and reduce bias due to differences in baseline characteristics between the exposed and unexposed groups. There were several important baseline imbalances in the exposed and unexposed groups, with infants who received early prostacyclin having more severe disease. Additionally, the finding of early prostacyclin use being associated with increased mortality in the full cohort but not in the matched cohort further suggests infants who received early prostacyclin were generally sicker and thus more likely to receive ECLS and have higher mortality. As infants who receive early prostacyclin therapy are more likely to receive ECLS, the finding of a decrease in odds of ECLS use to 40% of the baseline odds may suggest a meaningful reduction.

While the variables used in propensity score matching were selected based on previously described risk factors associated with ECLS in infants with CDH, there are missing variables that may affect the decision for ECLS use and outcomes, such as arterial partial pressure of oxygen, oxygen index, degree of hypoxemia, ventilator settings, use of vasopressors for blood pressure support, presence of cardiac dysfunction (which was excluded due to incomplete documentation), and presence of other comorbidities. Furthermore, propensity matched analysis can only identify patients similar to the treatment group and adjust for measured confounders. The consequences of unmeasured confounders remain difficult to ascertain. The E-values for receipt of ECLS and categorical ECLS duration were 2.58 and 2.42, respectively, suggesting that only a relatively small effect size of an unmeasured confounder would be needed to fully explain away the observed association in this study. Also interesting, based on the finding of an ICC of 0.24 with ECLS use, is that one-quarter of the total variation in whether an infant received ECLS depended upon the institution providing care after accounting for the other factors in the model, indicating differences between hospitals. Future randomized controlled trials (RCTs) may better evaluate the benefits of early prostacyclin therapy in infants with CDH-PH.

## EBM lesson: E-value

RCTs are the gold standard for measuring the effect of an intervention or treatment. Randomization minimizes potential measured (observed) and unmeasured (unobserved) confounders by balancing participants’ characteristics between the treatment and control groups, which allows attribution of differences in outcomes to the treatment [13]. However, sometimes RCTs in clinical settings are unavailable, and the best available data are from observational studies. One major limitation of observational studies is the potential for confounding, which results from differences between treatment groups that also affect the groups’ outcomes [13].

Propensity score matching helps to eliminate differences in baseline characteristics by identifying a cohort of participants for the control group similar to the participants in the treatment group through matching. This minimizes imbalance in measured confounders, but it does not account for potential unmeasured confounders [13]. The E-value was introduced to help assess the potential effect of unmeasured confounding on observed treatment-outcome associations. The E-value represents the “minimum strength of association on the risk ratio scale that an unmeasured confounder would need to have with both the treatment and outcome to fully explain away a specific treatment-outcome association, conditional on the measured covariates” [14]. In other words, the E-value reports how strong unmeasured confounders would need to be in order to produce the observed treatment-outcome association.

The E-value can be calculated as E-value = RR + $\sqrt{{{{{{\rm{RRx}}}}}} \, ({{{{{\rm{RR}}}}}}-1)}$, where RR is the observed risk ratio [14]. A small E-value implies that little unmeasured confounding is needed to explain the observed association (i.e., the RR observed is easily confounded) whereas a large E-value implies that the relationship is less easily confounded. The lowest E-value is 1, which suggests that no unmeasured confounder is needed to negate the observed treatment-outcome association [14]. In this study, Ramaraj et al. reported early prostacyclin therapy is associated with decreased ECLS use (23.1% with early prostacyclin therapy vs 42.9% without prostacyclin therapy) with an aOR of 0.39 (95% CI, 0.22–0.68) and E-value of 2.58. This means that an unmeasured confounder associated with both the treatment and outcome would need to have a relative risk ratio of at least 2.58 to explain away the observed association between early prostacyclin therapy and ECLS use reported in this study.

There are important limitations of E-values. First, E-values do not offer any insights into what the unmeasured confounders might be, the magnitude of effect the confounders might have on the observed association, or how to best handle potential confounding [15]. Second, E-values do not address other potential major biases that may play a role in observational studies, such as selection bias, attrition bias, missing data, and measurement error [14, 15]. Lastly, there is no current proposed threshold cutoff, like with the p-value, that defines a small versus large E-value to help readers interpret whether study inferences are likely confounded [15].
